# Body composition, physical fitness and cardiovascular risk factors in 9-year-old children

**DOI:** 10.1038/s41598-022-06578-w

**Published:** 2022-02-17

**Authors:** Pontus Henriksson, Johanna Sandborg, Maria Henström, Christine Delisle Nyström, Evelina Ek, Francisco B. Ortega, Marie Löf

**Affiliations:** 1 Department of Health, Medicine and Caring Sciences, Linköping University, Linköping, 58183 Sweden; 2 Department of Biosciences and Nutrition, Karolinska Institutet, Huddinge, Stockholm, Sweden; 3 PROFITH (PROmoting FITness and Health Through Physical Activity) Research Group, Department of Physical Education and Sports, Faculty of Sport Sciences, Research Institute of Sport and Health, University of Granada, Granada, Spain; 4 Faculty of Sport and Health Sciences, University of Jyväskylä, Jyväskylä, Finland

**Keywords:** Disease prevention, Nutrition, Paediatrics, Public health

## Abstract

The independent associations of body composition and physical fitness components with cardiovascular disease (CVD) risk factors in childhood are not fully understood. Thus, this cross-sectional study examined the independent associations of body composition and physical fitness with CVD risk factors in Swedish 9-year-old children (n = 411). Unadjusted linear regression analyses showed that body mass index (BMI), % fat mass and fat mass index were all positively associated with systolic and diastolic blood pressure, Homeostatic Model Assessment of Insulin Resistance (HOMA-IR) and Metabolic Syndrome (MetS) score (all β ≥ 0.229, *P* ≤ 0.001). These associations were virtually unaffected by adjustments for basic covariates (child’s age and sex, maternal educational level and maternal BMI), fat-free mass and physical fitness. Fat-free mass index had generally weak associations with CVD risk factors and no associations were statistically significant after adjustments (all *P* > 0.27). Greater cardiorespiratory fitness and motor fitness were associated with lower HOMA-IR and MetS score in unadjusted models (all β ≤ − 0.158, *P* ≤ 0.039) but not after adjustments for basic covariates and body composition. These findings indicate that cardiovascular health promotion in childhood may focus on the maintenance of a healthy fat mass.

## Introduction

Childhood obesity is a global public health challenge, and more than 300 million children have obesity (≥ 100 million) or overweight (≥ 200 million)^1^. Obesity in childhood is linked to several negative health outcomes later in life including an increased risk of later premature mortality^2,3^, disability^4^ and cardiovascular disease (CVD)^3,5,6^. There is also accumulating evidence that obesity is associated with impaired cardiovascular health already in childhood. For instance, previous studies have reported linear associations of body mass index (BMI) with greater cardiometabolic risk, Homeostatic Model Assessment of Insulin Resistance (HOMA-IR) and Metabolic Syndrome (MetS) scores in children and youth^7,8^. However, BMI is a relatively poor proxy of body composition especially in childhood^9^ and cannot differentiate between fat mass and fat-free mass^10^, which may have different effects on health outcomes^11^. Although studies have consistently shown positive associations of greater adiposity and fat mass with increased CVD risk factors in children^12–15^, few studies have examined the joint associations of accurately measured fat mass and fat-free mass with CVD risk factors. In particular, the associations of fat-free mass with CVD risk factors in children are poorly examined.

Physical fitness and in particular cardiorespiratory fitness has been associated with a more favorable CVD risk profile already in childhood^16,17^ and there is some evidence that high fitness may attenuate the negative effects of obesity on later cardiovascular health^6,18^. However, it is not fully examined whether the associations of physical fitness are influenced by accounting for accurately measured body composition (i.e., fat mass and fat-free mass) and vice versa. This is of importance as several studies have shown that body composition and physical fitness (especially when measured as performance in field-tests such as the 20 m shuttle run) are closely related to each other^19–22^. Therefore, the aim of this study was to investigate the independent associations of body composition and physical fitness with risk factors for cardiovascular disease (i.e. blood pressure, HOMA-IR and a composite MetS score) in 9-year-old children.

## Methods

### Study design and participants

This cross-sectional study used data from the SPINACH (Studies of Prospective health determinants in INfancy And CHildhood) study which utilized follow-up measurements conducted in 9-year-olds that had previously taken part in one of three previous studies: two birth cohorts^23,24^ and one randomized controlled trial in 4.5-year-old-children^25^ that all sampled participants from the general population of Östergötland, Sweden. The inclusion criteria for the birth cohorts were a healthy, full-term (born after at least 37 weeks of gestation) singleton infant^23,24^. The inclusion criteria for the randomized controlled trial was a healthy 4-year-old child that could be measured at 4.5 years of age (± 2 months). Exclusion criteria included if the child was diagnosed with a disease/disorder that would affect body size or feeding and if the parents had a serious physical or psychological disease making the study too demanding for the family^25^. The follow-up at 9 years of age in these three studies were coordinated and conducted during 2016–2020, and thus included identical measures of body composition, physical fitness and health outcomes. Of the 632 children originally measured in infancy and preschool-age, 411 children participated in the follow-up measurements at 9 years of age and were included in the study.

Participants were also invited to provide a blood sample for the analyses of additional CVD risk factors (i.e. HOMA-IR, components of MetS) and 175 children provided a fasting blood sample. The 175 children who provided a blood sample were fairly comparable to the 236 children that did not, with regard to average BMI (17.0 vs. 16.9 kg/m^2^), age (9.6 vs. 9.5 years), sex distribution (55.1% vs. 46.6% boys) as well as maternal educational attainment (80.5% vs. 75.0% university degree). Ethical permission was approved by the ethics committees in Linköping (ref no 2016/300-31) and Stockholm (ref no 2018/2220-32) and was conducted in accordance with the Declaration of Helsinki. All parents provided informed written consent.

### Body composition

Body composition and anthropometric measures were taken before the physical fitness tests, in accordance with standard procedures, i.e., wearing only underwear and no shoes^26^. Height was measured using a wall stadiometer (Tillquist, Spånga, Sweden) to the nearest 0.5 cm. Body composition was measured after a 2 h fast using air-displacement plethysmography (BodPod, COSMED USA, Concord, CA, USA), which is a reliable, valid and safe technique for the evaluation of body composition in children^26^. Briefly, body weight was measured to the nearest gram with an electronic scale attached to the air-displacement plethysmograph (BodPod, COSMED USA, Concord, CA, USA). Subsequently, body volume was measured using the BodPod and body density was calculated as measured weight divided by measured volume. Subsequently, body fatness was calculated utilizing the densities of fat- and fat-free mass according to Lohman’s model^27^. BMI was calculated as body weight (kg) divided by height (m) squared. Overweight and obesity were classified according to the BMI cut-offs by Cole and Lobstein^28^. Fat mass index was calculated by dividing fat mass (kg) with height (m) squared (i.e., fat mass/height^2^), whereas fat-free mass index was calculated as the amount of fat-free mass (kg) divided by height (m) squared (i.e., fat-free mass/height^2^).

### Physical fitness

Physical fitness was measured using the ALPHA health related fitness test-battery^29^. The ALPHA-battery of tests are considered valid and reliable for evaluation of physical fitness levels in children and adolescents aged 6–18 years and detailed information about the ALPHA battery is available elsewhere^29^. Cardiorespiratory fitness was assessed using the 20-m shuttle run test^29,30^ and this was conducted last considering the maximality of the test. The participant started at a pace of 8.5 km/h, increasing with 0.5 km/h each minute of the test while running between two lines 20 m apart and keeping pace with the pre-recorded audio signals. The test continued until the child decided to stop due to fatigue or if the participant failed to run the 20-m distance before the signal was played two consecutive times.

Upper body muscular strength was evaluated using the hand grip strength test with an analogue dynamometer (TKK 5001, Grip-A, Takei, Tokyo, Japan). Hand size of the participant was measured to the nearest 0.5 cm, and the grip span of the dynamometer was adjusted in order to assure the correct setting to acquire maximal hand grip strength^31^. The participant squeezed the dynamometer with maximal power two times in each hand and the best of two attempts was registered. Thereafter, the average of both hands was calculated and used in the analyses.

Lower body muscular strength was assessed using the best of two attempts of the standing long jump test, where the participant jumped as far as possible with both feet together while remaining upright^29^. The distance from the start-line to the back of the heel nearest to the starting point was measured.

Motor fitness was evaluated using the 4 × 10 m shuttle run test^29^. During the test, the child ran as fast as possible between two parallel lines 10 m apart. This test was performed twice with a total distance of 40 m (4 × 10 m) covered each time. Since lower scores (seconds) indicate higher performance, the results were inverted (by multiplying by − 1) in the statistical analyses.

### Cardiovascular risk factors

Systolic and diastolic blood pressure were measured in millimeter of mercury (mmHg) with an electric sphygmomanometer (WelchAllyn, ProBP 3400 series, NY, USA). The participants were sitting in an upright resting position and had to rest at least five minutes before the measurement. Two readings of blood pressure were done; if the readings differed by more than 10 mmHg, a third measurement was carried out. The mean value of two or three measures of blood pressure was calculated and used in the analyses.

Children provided fasting venous blood samples which were analyzed for glucose, insulin, total cholesterol, high density lipoprotein cholesterol (HDL cholesterol) and triglycerides as described previously^32^.

All analyses were conducted at the Department of Clinical Chemistry, Linköping University, Linköping, Sweden, which is accredited for the analyses (ISO/IEC 17025).

 To estimate insulin resistance, HOMA-IR was calculated as fasting insulin [µU/L] × fasting glucose [mmol/L])/22.5^33^. Due to the skewed distribution of HOMA-IR, values were transformed with the natural logarithm (ln) in the statistical analyses. A continuous cardiovascular risk score based on the most used definition of the metabolic syndrome (MetS) was used in accordance with previous studies^7,32^. Since body composition was an independent variable in the analysis, waist circumference was not included in the score. Thus, the MetS score was calculated as the normalized sum of sex-specific z-scores for triglycerides, inverted (by multiplying with − 1) HDL-cholesterol, fasting glucose and the average of systolic and diastolic blood pressure as described previously^7,32^.

### Statistical analyses

Linear regression was utilized in the statistical analyses. First, we examined the associations of body composition and physical fitness using one unadjusted model and one model that was adjusted for the child’s age and sex, maternal educational level and maternal BMI. Additionally, models with fat mass index and fat-free mass index as the independent variable were mutually adjusted for each other. Second, we examined associations of body composition and physical fitness with CVD risk factors and three linear regression models were created: (1) unadjusted model; (2) basic adjusted model including child’s sex and age at measurement, maternal BMI and maternal educational attainment as covariates (models with fat mass index and fat-free mass index as the independent variable were also mutually adjusted for each other); (3) basic adjusted model plus cardiorespiratory fitness and handgrip strength (where body composition was the exposure) or adjusted model plus fat mass index and fat-free mass index (where physical fitness measures were the exposure). Thus, the latter model provides estimates that are mutually adjusted for body composition and cardiorespiratory fitness. Furthermore, we observed no violations against the assumptions of the regression models^34^. The statistical analyses were conducted using SPSS Statistics (IMB SPSS statistics, version 26, IBM Corp., NY, USA) and *P* < 0.05 was regarded as statistically significant.

### Ethics approval and consent to participate

Ethical permission for all studies in this manuscript was approved by the ethics committees in Linköping (ref no. 2016/300-31) and Stockholm (ref no 2018/2220-32). All parents provided informed written consent before any measurements were conducted.

## Results

### Descriptive statistics

Table 1 describes the 411 children (200 girls and 211 boys) who participated in this study. They were on average 9.5 years (SD 0.1) old at the time of the measurement with an average height and weight of 139.4 cm (SD 6.1) and 33.2 kg (SD 6.8), respectively. Of the 411 children, 10.9% (22 girls and 23 boys) were classified as having overweight and another 1.9% (4 girls and 4 boys) were classified as having obesity. Maternal age was on average 40.7 years (SD 3.9) and average BMI was 24.6 kg/m^2^ (SD 4.1, n = 400). The majority of mothers had a university degree (78.1%, n = 321), whereas 21.7% (n = 89) had a high school education and 0.2% (n = 1) had a primary school (9 years) education.

**Table 1:** Descriptive characteristics of the 9-year-old children.

	All		Girls		Boys	
	n	Value	n	Value	n	Value
**General characteristics**						
Age (years)	411	9.5 (0.1)	200	9.5 (0.1)	211	9.5 (0.1)
Height (cm)	411	139.4 (6.1)	200	138.7 (5.9)	211	140.2 (6.2)
Weight (kg)	411	33.2 (6.8)	200	32.7 (6.8)	211	33.6 (6.7)
**Body composition**						
BMI (kg/m^2^)	411	16.9 (2.5)	200	16.9 (2.6)	211	17.0 (2.4)
FM (%)	410	19.7 (7.5)	200	21.2 (7.3)	210	18.3 (7.3)
FMI (kg/m^2)^	410	3.5 (1.9)	200	3.7 (2.0)	210	3.3 (1.9)
FFMI (kg/m^2^)	410	13.5 (1.1)	200	13.2 (1.0)	210	13.8 (1.1)
**Physical fitness**						
20 m shuttle run (laps)	404	20.6 (9.8)	196	18.1 (7.4)	208	23.0 (11.2)
Handgrip strength (kg)	409	16.7 (3.1)	199	15.8 (2.8)	210	17.4 (3.1)
Standing long jump (cm)	409	124 (19)	199	118 (18)	210	129 (19)
4 × 10 m shuttle run (s)	407	14.0 (1.1)	196	14.2 (1.0)	211	13.8 (1.1)
**CVD risk factors**						
Systolic BP (mmHg)	409	111 (9)	198	111 (10)	211	110 (9)
Diastolic BP (mmHg)	409	69 (6)	198	69 (6)	211	68 (6)
Glucose (mmol/l)^a^	173	5.1 (0.4)	92	5.0 (0.4)	81	5.1 (0.4)
Insulin (mIE/l)^b^	169	7.4 (3.8)	89	8.1 (4.0)	80	6.7 (3.5)
HOMA-IR	166	1.7 (0.9)	87	1.8 (1.0)	79	1.5 (0.8)
Total cholesterol (mmol/l)^a^	175	4.2 (0.7)	94	4.3 (0.6)	81	4.1 (0.7)
LDL cholesterol (mmol/l)^a^	175	2.2 (0.6)	94	2.3 (0.6)	81	2.1 (0.6)
HDL cholesterol (mmol/l)^a^	175	1.7 (0.4)	94	1.7 (0.4)	81	1.7 (0.3)
Triglycerides (mmol/l)^a^	175	0.6 (0.2)	94	0.7 (0.2)	81	0.6 (0.2)
MetS score (Z-score)	173	0.0 (1.0)	92	0.0 (1.0)	81	0.0 (1.0)

Values are presented as means (standard deviations). BMI, body mass index; BP, blood pressure; FM, fat-mass; FMI, fat-mass index; FFMI, fat-free mass index; CVD risk factors, cardiovascular disease risk factors; HDL, high density lipoprotein cholesterol; LDL, low density lipoprotein cholesterol; HOMA-IR, Homeostatic Model Assessment of Insulin Resistance; MetS score, metabolic syndrome score calculated using normalized sum of sex-specific z-scores for triglycerides, inverted (by multiplying with − 1), HDL-cholesterol, fasting glucose and the average of systolic and diastolic blood pressure.

^a^Measured in plasma.

^b^Measured in serum.

### Associations of body composition with physical fitness

Table 2 displays the associations of body composition with physical fitness. Higher BMI and % fat mass were associated with poorer physical fitness in the weight-bearing fitness test (i.e., cardiorespiratory fitness, lower body strength and motor fitness) (all β ≤ − 0.219, all *P* ≤ 0.001) but with better performance in upper-body muscular strength (i.e., handgrip strength, β ≥ 0.131, all *P* ≤ 0.008). Fat mass index and fat-free mass index had joint but opposite associations with physical fitness for the weight-bearing fitness tests in the adjusted models. Thus, higher fat mass index was associated with poorer cardiorespiratory fitness, lower body strength and motor fitness (all β ≤ − 0.439, all *P* ≤ 0.001), whereas a higher fat-free mass index was associated with better performance in the aforementioned physical fitness dimensions (all β ≥ 0.141, all *P* ≤ 0.005). Both fat mass index and fat-free mass index were positively associated with upper body muscular strength, although associations were considerably stronger for fat mass index as compared with fat-free mass index (β = 0.495 vs. 0.144 in the adjusted model).

**Table 2:** Associations of body composition with physical fitness in 9-year-old children.

Body composition (x)	Fitness variables (y)	Unadjusted		Adjusted^a^	
		β	*P*	β	*P*
BMI (kg/m^2^)	Cardiorespiratory fitness	− 0.307	< 0.001	− 0.306	< 0.001
	Upper body strength	0.408	< 0.001	0.467	< 0.001
	Lower body strength	− 0.243	< 0.001	− 0.219	< 0.001
	Motor fitness^b^	− 0.235	< 0.001	− 0.224	< 0.001
FM (%)	Cardiorespiratory fitness	− 0.470	< 0.001	− 0.431	< 0.001
	Upper body strength	0.131	0.008	0.209	< 0.001
	Lower body strength	− 0.452	< 0.001	− 0.402	< 0.001
	Motor fitness^b^	− 0.415	< 0.001	− 0.385	< 0.001
FMI (kg/m^2^)	Cardiorespiratory fitness	− 0.425	< 0.001	− 0.439	< 0.001
	Upper body strength	0.223	< 0.001	0.144	0.002
	Lower body strength	− 0.403	< 0.001	− 0.446	< 0.001
	Motor fitness^b^	− 0.384	< 0.001	− 0.452	< 0.001
FFMI (kg/m^2^)	Cardiorespiratory fitness	0.040	0.42	0.141	0.005
	Upper body strength	0.537	< 0.001	0.495	< 0.001
	Lower body strength	0.150	0.002	0.278	< 0.001
	Motor fitness^b^	0.139	0.005	0.286	< 0.001

β, standardized regression coefficient; BMI, body mass index; FFMI, fat-free mass index; FM, fat mass; FMI, fat mass index.

^a^Adjusted for child’s age and sex, maternal educational level and maternal BMI (for models with FMI and FFMI as independent variables, the models were also mutually adjusted for FMI and FFMI).

^b^Since lower scores indicate higher performance, results are inverted.

### Associations of body composition with cardiovascular risk factors

Associations of body composition with cardiovascular risk factors are presented in Table 3. BMI, % fat mass and fat mass index were positively associated with the cardiovascular risk factors (all β ≥ 0.198, all *P* ≤ 0.002) in all three regression models. Fat-free mass index had weak, but statistically significant associations with systolic blood pressure and MetS score in the unadjusted models (β ≥ 0.170, all *P* ≤ 0.026) although these associations became weaker in the basic adjusted model (adjusted child’s age and sex, maternal educational attainment and BMI) and not statistically significant (*P* ≥ 0.27) after further adjustments for physical fitness.

**Table 3:** Associations of body composition with cardiovascular risk factors in 9-year-old children.

Body composition (x)	CVD risk factors (y)	Unadjusted		Basic adjustment^a^		Basic adjustment^a^ + fitness^b^	
		β	*P*	β	*P*	β	*P*
BMI (kg/m^2^)	Systolic BP	0.311	< 0.001	0.293	< 0.001	0.240	< 0.001
	Diastolic BP	0.229	< 0.001	0.198	< 0.001	0.204	0.001
	HOMA-IR	0.409	< 0.001	0.341	< 0.001	0.292	0.002
	MetS score	0.430	< 0.001	0.381	< 0.001	0.334	< 0.001
FM (%)	Systolic BP	0.295	< 0.001	0.263	< 0.001	0.248	< 0.001
	Diastolic BP	0.240	< 0.001	0.201	< 0.001	0.231	< 0.001
	HOMA-IR	0.478	< 0.001	0.399	< 0.001	0.339	< 0.001
	MetS score	0.389	< 0.001	0.349	< 0.001	0.312	< 0.001
FMI (kg/m^2^)	Systolic BP	0.288	< 0.001	0.215	< 0.001	0.206	< 0.001
	Diastolic BP	0.241	< 0.001	0.201	< 0.001	0.231	< 0.001
	HOMA-IR	0.494	< 0.001	0.432	< 0.001	0.375	< 0.001
	MetS score	0.442	< 0.001	0.388	< 0.001	0.368	< 0.001
FFMI (kg/m^2^)	Systolic BP	0.201	< 0.001	0.140	0.011	0.069	0.27
	Diastolic BP	0.094	0.059	0.018	0.75	− 0.028	0.66
	HOMA-IR	0.050	0.52	− 0.046	0.55	− 0.088	0.33
	MetS score	0.170	0.026	0.065	0.39	− 0.015	0.87

β, standardized regression coefficient; BP, blood pressure; BMI, body mass index; CVD risk factors, cardiovascular disease risk factors; FFMI, fat-free mass index; FM, fat mass; FMI, fat-mass index; HOMA-IR, Homeostatic Model Assessment of Insulin Resistance; MetS score, metabolic syndrome score.

^a^Adjusted for child’s age and sex, maternal educational level and maternal BMI (for models with FMI and FFMI as independent variables, the models were also mutually adjusted for FMI and FFMI).

^b^Additionally adjusted for cardiorespiratory fitness (20 m shuttle run) and handgrip strength.

### Associations of physical fitness with cardiovascular risk factors

Table 4 presents the associations of physical fitness with cardiovascular risk factors. Higher cardiorespiratory fitness and motor fitness were associated lower HOMA-IR and MetS scores in the unadjusted model (β ≤ − 0.158, *P* ≤ 0.039). However, estimates were attenuated in the basic adjusted model and were not statistically significant (*P* ≥ 0.28) in the third model (adjusting for basic covariates plus body composition). Greater upper-body strength was associated with higher systolic and diastolic blood pressure as well as MetS score in the unadjusted model (β ≥ 0.108, all *P* ≤ 0.030). Although estimates were weakened by adjustments, upper-body strength remained positively associated with systolic blood pressure β = 0.129, all *P* = 0.029) after adjustments for basic covariates and body composition. Lower body strength was inversely associated with HOMA-IR in all three regression models (β ≤ − 0.160, all *P* ≤ 0.047) although associations were attenuated by adjustments.

**Table 4:** Associations of physical fitness with cardiovascular risk factors in 9-year-old children.

Fitness variables (x)	CVD risk factors (y)	Unadjusted		Basic adjustment^a^		Basic adjustment^a^ + body composition^b^	
		β	*P*	β	*P*	β	*P*
Cardiorespiratory fitness	Systolic BP	− 0.081	0.11	− 0.046	0.39	0.039	0.48
	Diastolic BP	− 0.028	0.57	0.013	0.80	0.103	0.070
	HOMA-IR	− 0.303	< 0.001	− 0.216	0.008	− 0.088	0.28
	MetS score	− 0.158	0.039	− 0.108	0.19	0.031	0.71
Upper body strength	Systolic BP	0.198	< 0.001	0.221	< 0.001	0.129	0.029
	Diastolic BP	0.108	0.030	0.122	0.017	0.080	0.18
	HOMA-IR	0.057	0.46	0.123	0.12	0.070	0.42
	MetS score	0.188	0.013	0.229	0.003	0.144	0.10
Lower body strength	Systolic BP	− 0.055	0.27	− 0.006	0.91	0.055	0.33
	Diastolic BP	− 0.082	0.098	− 0.039	0.45	0.028	0.62
	HOMA-IR	− 0.320	< 0.001	− 0.264	< 0.001	− 0.160	0.047
	MetS score	− 0.129	0.092	− 0.079	0.32	0.017	0.83
Motor fitness^c^	Systolic BP	− 0.051	0.31	− 0.008	0.88	0.065	0.23
	Diastolic BP	− 0.106	0.032	− 0.068	0.18	0.003	0.96
	HOMA-IR	− 0.275	< 0.001	− 0.165	0.038	− 0.031	0.69
	MetS score	− 0.249	< 0.001	− 0.186	0.019	− 0.080	0.32

β, standardized regression coefficient; BP, blood pressure; CVD risk factors, cardiovascular disease risk factors; HOMA-IR, Homeostatic Model Assessment of Insulin Resistance; MetS score, metabolic syndrome score.

^a^Adjusted for child’s age and sex, maternal educational level and maternal BMI.

^b^Additionally adjusted for fat-mass index and fat-free mass index.

^c^Since lower scores indicate higher performance, results are inverted.

### Sensitivity analyses

Although data collection at the 9-year follow-up, which is the focus of this paper, was carefully standardized, children were originally from three previous studies. Hence, we re-analyzed the associations of body composition and physical fitness with the MetS score only using data from MINISTOP^25^, which is the largest individual study (Tables S1 and S2). We also re-analyzed the data using only data from the two birth cohort studies (Tables S1 and S2). As shown in the tables, associations were generally very comparable in terms of direction and magnitude when analyzing all participants or participants from MINISTOP or the birth cohorts separately. Furthermore, since it has been suggested that normalizing body mass and composition (i.e., fat and fat-free mass) with height squared (i.e., the calculation of BMI, fat mass index and fat-free mass index) may not be sufficient in children^15^, we performed an additional analysis where height was added in the final model. As shown in Table S3, this adjustment had minor influence on the estimates, and results remained virtually the same. Finally, we also examined the influence of expressing cardiorespiratory fitness in estimated VO_2_-max using the equation by Léger et al.^30^ as shown in Table S4. However, estimates were very comparable to our main analysis, i.e., cardiorespiratory fitness was inversely associated with HOMA-IR and the MetS score but associations were attenuated by adjustments for covariates and body composition.

## Discussion

### Principal findings

This study comprehensively examined associations of body composition and physical fitness with CVD risk factors in 9-year-old children and reports several findings of interest. First, body composition and physical fitness were strongly associated, which highlight the need of mutual adjustments when examining the independent associations of body composition and physical fitness with CVD risk factors. Second, accurately measured fat mass and fat mass index were strongly and positively associated with CVD risk factors also after adjustments for covariates including physical fitness. Third, accurately measured fat-free mass index had generally weak associations with CVD risk factors that were attenuated after adjustments. Finally, greater cardiorespiratory fitness and motor fitness were associated with lower HOMA-IR and MetS score, although associations were strongly attenuated by the adjustments for other covariates including body composition (fat mass index and fat-free mass index).

### Body composition

We found that body composition was strongly associated with physical fitness which extends our previous findings in preschool aged children^19^ and previous studies in children that have not examined the combined association of fat mass and fat-free mass on physical fitness. Previous studies have consistently reported that greater fat mass is strongly associated with CVD risk factors such as higher blood pressure, LDL cholesterol, triglycerides and insulin values as well as lower HDL cholesterol^12–15^. Our results confirm these findings and also report that the associations of BMI, % fat mass and fat mass index were quite unaffected by adjustments for covariates including physical fitness and fat-free mass index (only the model with fat mass index). There are many mechanisms by which obesity may increase the prevalence of CVD risk factors or actual CVD events^35,36^. For instance, impaired adipogenesis, altered fat deposition, inflammatory and adipokine dysregulation, adipose tissue hypoxia, increased circulating free fatty acids, oxidative stress and lipotoxicity have been suggested as relevant pathways^35^. Furthermore, excess body fatness is closely connected with insulin resistance which in turn is related with dyslipidemia (i.e., high levels of triglycerides and low levels of HDL cholesterol)^36^. Finally, obesity induce adaptations in the cardiac system including increased cardiac output and systemic vascular resistance which elevate the blood pressure^37^.

Relatively few studies have examined associations between fat-free mass with CVD risk factors in children. Grijalva-Eternod et al.^38^ reported that higher fat-free mass normalized for height was associated with higher systolic, but not diastolic blood pressure, which agree with our findings in the unadjusted and adjusted models. Interestingly, associations were attenuated and not statistically significant after accounting for physical fitness (i.e., cardiorespiratory fitness and handgrip strength) in our analyses. Another study reported no associations with fat-free mass and insulin resistance in 7–9-year-old children although a positive association was observed in girls only after the age of 10^15^ which may be reconciled with our findings. Thus, based on our and the previous studies^15,38,39^, fat-free mass appears not to have any beneficial association with CVD risk factors.

### Physical fitness

Greater cardiorespiratory fitness and motor fitness in our unadjusted analyses were associated with lower HOMA-IR and MetS score which generally agrees well with previous studies of fitness and CVD risk factors^40–46^. However, previous studies have generally shown that the associations of cardiorespiratory fitness with CVD risk factors are strongly attenuated by adjustments for BMI or body fatness, both in studies that have used laboratory^47,48^ or field measures^44–46^ of cardiorespiratory fitness. Data from this and previous studies ^19,20,22^ have shown that performance in the 20 m shuttle run is strongly and inversely associated with body fatness in children. Given the close relationship between cardiorespiratory fitness and body fatness, it is possible that the unadjusted and statistically significant associations of cardiorespiratory and motor fitness with lower HOMA-IR and MetS are due to differences in body composition (and especially a higher fat mass). This would explain why these estimates are no longer statistically significant in the models that accounted for fat mass and fat-free mass and highlight the importance of considering body composition when examining associations of physical fitness and health outcomes. Although cardiorespiratory and motor fitness are regarded as markers of health^49,50^, it is important to consider that associations between fitness and health outcomes may, at least partly, be due to other factors such as body fatness.

We observed that upper body strength and lower body strength in some cases had opposite associations with the measured CVD risk factors. For instance, greater upper body strength was associated with higher systolic blood pressure and MetS score, whereas greater lower body strength was associated with lower HOMA-IR and MetS score. These results may be reconciled with previous studies of muscular strength and CVD risk factors in youth. Such studies have generally reported positive or null associations with handgrip strength^46,51,52^ and inverse or null associations with standing long jump^46,51,52^ in analyses that did not account for body composition or fatness. As shown in Table 2, associations between fat mass and upper body and lower body strength differed, which may be due to the nature of the fitness tests (weight bearing or not). Although greater fat-free mass was associated with better performance in both tests, greater fat mass was associated with better performance in the handgrip strength (non-weight bearing), but with poorer performance in the standing long jump (weight bearing). Considering the strong associations of fat mass with CVD risk factors, this difference may at least partly account for the difference in associations between strength and CVD risk factors. Indeed, when additionally adjusting for fat mass and fat-free mass, associations were generally strongly attenuated, although handgrip strength remained positively associated with systolic blood pressure and lower body strength inversely associated with HOMA-IR. Thus, future studies should consider adjustments for body composition to elucidate the independent associations of physical fitness with CVD risk factors.

### Strengths and limitations

A major strength of this study was the relatively large sample of children (n = 411) that were measured using accurate body composition methodology (air-displacement plethysmography)^26^. Furthermore, we were able to analyze the joint associations of body composition (fat mass and fat-free mass) and physical fitness with CVD risk factors. Another strength is that children were measured at 9.5 years of age within a narrow time frame (SD 0.1 years).

The current study also has some limitations that needs to be considered. First, the cross-sectional study design limits interferences regarding the casualty of the examined associations. Moreover, the high proportion of mothers with a university degree may influence generalizability of the results. However, maternal educational attainment was accounted for in the analyses and had little influence on the estimates. Furthermore, we lacked data regarding pubertal status in the analyses which is a limitation. According to a large Danish study^53^, less than 2% of boys and girls had reached Tanner stage 2 at 9.5 years of age and virtually no one had reached stage 3, 4 or 5 at that time point. Thus, we consider it likely that the effects of puberty have limited influence on our estimates, although further studies should consider accounting for pubertal status in the analysis.

### Implications

The findings of our study have some implications that may be of importance for the promotion of cardiovascular health in children. First, our findings showed that fat mass index and fat-free mass index have generally independent but opposite associations with physical fitness, which indicate that analyses that include both body composition and physical fitness variables should be mutually adjusted for each other. Second, BMI was generally as strongly associated to CVD risk factors as accurately measured fat mass, which indicate that more advanced body composition measurements (e.g., air-displacement plethysmography) may not convey cardiovascular health better than BMI, which is easily measured. Third, fat-free mass had no beneficial associations with CVD risk factors which provides support that cardiovascular health promotion in childhood should focus on the maintenance of a healthy fat mass. Finally, higher cardiorespiratory fitness and motor fitness were associated with lower HOMA-IR and MetS score. However, associations were strongly attenuated by accounting for body composition (fat mass and fat-free mass). Nevertheless, evidence from longitudinal studies in childhood and adolescence suggest that greater cardiorespiratory fitness, independent of BMI, is associated with better cardiovascular outcomes later in life^2,6,18,54^. Thus, further studies are needed to elucidate the independent influence of physical fitness on cardiovascular health in childhood and adolescence.

## Conclusion

In conclusion, greater BMI and fat mass was associated with CVD risk factors even after adjustments for covariates and physical fitness. Importantly, associations with BMI were generally as strong as with accurately measured fat mass which may have implication given the easy measurement of BMI in children. However, fat-free mass did not have any beneficial associations with CVD risk factors which support the notion that the focus for cardiovascular health promotion during childhood could be on excess fat mass and not on the fat-free mass. Finally, higher cardiorespiratory and motor fitness were associated with lower HOMA-IR and MetS score although associations were strongly attenuated by adjusting the estimates for body composition (fat mass and fat-free mass).

## Supplementary Information

Supplementary Information.

## Data Availability

The datasets used and analyzed during the current study are available from the corresponding author on reasonable request.
