# Supplementary material for: Body composition, physical fitness and cardiovascular risk factors in 9-year-old children
Source: Sci Rep. 2022 Feb 17;12:2665. doi: 10.1038/s41598-022-06578-w (PMC8854391; doi:10.1038/s41598-022-06578-w)
Supplement: Supplementary file 1 — Supplementary Information. [file 41598_2022_6578_MOESM1_ESM.pdf]

Supplementary material

Body composition, physical fitness and cardiovascular risk factors in 9-year-old children

Pontus Henriksson* [1], Johanna Sandborg[1,2], Maria Henström[2], Christine Delisle Nyström[2], Evelina Ek[1], Francisco B Ortega[3,2,4], Marie Löf[1,2]

[1] Department of Health, Medicine and Caring Sciences, Linköping University, Linköping, Sweden.

[2] Department of Biosciences and Nutrition, Karolinska Institutet, Huddinge, Stockholm, Sweden.

[3] PROFITH (PROmoting FITness and Health through physical activity) research group, Department of Physical Education and Sports, Faculty of Sport Sciences, Research Institute of Sport and Health, University of Granada, Spain.

[4] Faculty of Sport and Health Sciences, University of Jyväskylä, Jyväskylä, Finland.

*** Corresponding author:** Pontus Henriksson, Department of Health, Medicine and Caring Sciences, Linköping University, 581 83 Linköping, Sweden. Telephone number: 013-28 10 00. E-mail: pontus.henriksson@liu.se

Table S1. Sensitivity analysis examining the associations of body composition with metabolic syndrome (MetS score) in 9-year-old children stratified by studies.

Body composition	Study	Unadjusted		Basic adjustment[a]		Basic adjustment[a] + fitness[b]	
		**β**	***P***	**β**	***P***	**B**	***P***
BMI (kg/m^2)	All participants (n = 173)	0.430	< 0.001	0.381	< 0.001	0.334	< 0.001
	Birth cohorts (n = 87)	0.343	0.001	0.330	0.006	0.292	0.060
	MINISTOP (n = 86)	0.500	< 0.001	0.448	< 0.001	0.438	< 0.001
FM (%)	All participants (n = 172)	0.389	< 0.001	0.349	< 0.001	0.312	< 0.001
	Birth cohorts (n = 87)	0.367	< 0.001	0.352	0.006	0.334	0.023
	MINISTOP (n = 85)	0.410	< 0.001	0.386	< 0.001	0.353	0.003
FMI (kg/m^2)	All participants (n = 172)	0.442	< 0.001	0.388	< 0.001	0.368	< 0.001
	Birth cohorts (n = 87)	0.398	< 0.001	0.401	0.002	0.388	0.010
	MINISTOP (n = 85)	0.479	< 0.001	0.409	< 0.001	0.412	0.001
FFMI (kg/m^2)	All participants (n = 172)	0.170	0.026	0.065	0.39	-0.015	0.87
	Birth cohorts (n = 87)	0.041	0.71	0.016	0.89	-0.064	0.64
	MINISTOP (n = 85)	0.289	0.007	0.105	0.33	0.050	0.70

β, standardized regression coefficient; BMI, body mass index; FFMI, fat-free mass index; FM, fat mass; FMI, fat-mass index.

[a] Adjusted for child's age and sex, maternal educational level and maternal BMI (for models with FMI and FFMI as independent variables, the models were also mutually adjusted for FMI and FFMI).

[b] Additionally adjusted for cardiorespiratory fitness (20 m shuttle run) and handgrip strength.

Table S2. Sensitivity analysis examining the associations of physical fitness with metabolic syndrome (MetS score) in 9-year-old children stratified by studies.

		Unadjusted		Basic adjustment[a]		Basic adjustment[a] + body composition[b]	
**Fitness variables**	**Study**	**β**	***P***	**β**	***P***	**β**	***P***
Cardiorespiratory fitness	All participants (n = 172)	-0.158	0.039	-0.108	0.19	0.031	0.71
	Birth cohorts (n = 86)	-0.156	0.15	-0.107	0.37	0.053	0.67
	MINISTOP (n =86)	-0.154	0.16	-0.095	0.40	0.056	0.62
Upper body strength	All participants (n = 173)	0.188	0.013	0.229	0.003	0.144	0.10
	Birth cohorts (n = 87)	0.169	0.12	0.189	0.095	0.139	0.30
	MINISTOP (n = 86)	0.202	0.062	0.244	0.024	0.106	0.39
Lower body strength	All participants (n = 173)	-0.129	0.092	-0.079	0.32	0.017	0.83
	Birth cohorts (n = 87)	-0.150	0.17	-0.114	0.32	-0.062	0.59
	MINISTOP (n = 86)	-0.109	0.32	-0.037	0.74	0.187	0.15
Motor fitness[c]	All participants (n = 173)	-0.249	0.001	-0.186	0.019	-0.080	0.32
	Birth cohorts (n = 87)	-0.167	0.12	-0.097	0.41	0.038	0.74
	MINISTOP (n = 86)	-0.314	0.003	-0.250	0.023	0.088	0.47

β, standardized regression coefficient.

[a] Adjusted for child's age and sex, maternal educational level and maternal BMI.

[b] Additionally adjusted for fat-mass index and fat-free mass index.

[c] Since lower scores indicate higher performance, results are inverted.

Table S3. Sensitivity analysis exploring the influence of additional adjustment for child's height on the associations of body composition with cardiovascular risk factors in 9-year-old children.

		Basic adjustment[a] + fitness[b]		Basic adjustment[a] + fitness[b] + height	
**Body composition (x)**	**CVD risk factors (y)**	**β**	***P***	**β**	***P***
BMI (kg/m^2)	Systolic BP	0.240	< 0.001	0.231	< 0.001
	Diastolic BP	0.204	0.001	0.205	< 0.001
	HOMA-IR	0.292	0.002	0.292	0.002
	MetS score	0.334	< 0.001	0.333	< 0.001
FM (%)	Systolic BP	0.248	< 0.001	0.238	< 0.001
	Diastolic BP	0.231	0.002	0.240	< 0.001
	HOMA-IR	0.339	< 0.001	0.329	< 0.001
	MetS score	0.312	< 0.001	0.305	< 0.001
FMI (kg/m^2)	Systolic BP	0.206	0.001	0.192	0.002
	Diastolic BP	0.231	< 0.001	0.241	< 0.001
	HOMA-IR	0.375	< 0.001	0.364	< 0.001
	MetS score	0.368	< 0.001	0.360	< 0.001
FFMI (kg/m^2)	Systolic BP	0.069	0.27	0.078	0.21
	Diastolic BP	-0.028	0.66	-0.034	0.59
	HOMA-IR	-0.088	0.33	-0.068	0.46
	MetS score	-0.015	0.87	-0.003	0.98

β, standardized regression coefficient; BP, blood pressure; BMI, body mass index; CVD risk factors, cardiovascular disease risk factors; FFMI, fat-free mass index; FM, fat mass; FMI, fat-mass index; HOMA-IR, Homeostatic Model Assessment of Insulin Resistance; MetS score, metabolic syndrome score.

[a] Adjusted for child's age and sex, maternal educational level and maternal BMI (for models with FMI and FFMI as independent variables, the models were also mutually adjusted for FMI and FFMI).

[b] Additionally adjusted for cardiorespiratory fitness (20 m shuttle run) and handgrip strength.

Table S4. Sensitivity analysis examining associations of cardiorespiratory fitness (expressed in VO_2max and laps) fitness with cardiovascular risk factors in 9-year-old children.

		Unadjusted		Basic adjustment[a]		Basic adjustment[a] + body composition[b]	
**Fitness variables (x)**	**CVD risk factors (y)**	**β**	***P***	**β**	***P***	**β**	***P***
Cardiorespiratory fitness ($V0_2$max)	Systolic BP	-0.069	0.17	-0.028	0.62	0.050	0.38
	Diastolic BP	-0.022	0.65	0.036	0.51	0.119	0.042
	HOMA-IR	-0.249	0.001	-0.173	0.047	-0.039	0.66
	MetS score	-0.142	0.064	-0.087	0.32	0.047	0.59
Cardiorespiratory fitness (laps, main analyses)	Systolic BP	-0.081	0.11	-0.046	0.39	0.039	0.48
	Diastolic BP	-0.028	0.57	0.013	0.80	0.103	0.070
	HOMA-IR	-0.303	< 0.001	-0.216	0.008	-0.088	0.28
	MetS score	-0.158	0.039	-0.108	0.19	0.031	0.71

β, standardized regression coefficient; BP, blood pressure; CVD risk factors, cardiovascular disease risk factors; HOMA-IR, Homeostatic Model Assessment of Insulin Resistance; MetS score, metabolic syndrome score.

[a] Adjusted for child's age and sex, maternal educational level and maternal BMI.

[b] Additionally adjusted for fat-mass index and fat-free mass index.
